# Awareness of the need for safe storage of Methadone at home is not improved by the use of protocols on recording information giving

**DOI:** 10.1186/1477-7517-5-15

**Published:** 2008-04-30

**Authors:** Annemarie Mullin, Rosanna J McAuley, Derrett J Watts, Ilana B Crome, Roger N Bloor

**Affiliations:** 1 School of Medicine, Keele University, Staffordshire, ST5 5BG, UK; 2 Edward Myers Unit, North Staffordshire Combined Healthcare NHS Trust, Stoke on Trent, Staffordshire, ST4 6TH. UK; 3 Academic Psychiatry Department, Keele University Medical School. Harplands Campus, Stoke on Trent, Staffordshire, ST4 6TH. UK

## Abstract

**Background:**

Methadone is a synthetic, narcotic analgesic used in the treatment of drug misuse. Tragedies involving children being poisoned by the accidental ingestion of methadone are no longer a rare occurrence. Following an audit of the effectiveness of the provision and recall of information to patients attending an NHS Methadone Clinic a protocol was introduced to ensure that staff documented the provision of such information and patients gave a written confirmation that they had received the information.

**Methods:**

The study was undertaken in the setting of an NHS methadone clinic with the aim of re- auditing the storage of methadone at home following the introduction of the new protocols. 174 patients completed an anonymous questionnaire regarding where they store methadone at home and whether they recall being given advice about safe storage. Community pharmacists were contacted by telephone to assess the level of advice given to methadone patients regarding safety.

**Results:**

Only 49 (28.2%) patients recalled being given advice about safe storage, 24 (13.8%) recalled that information was provided by clinic staff. 170 (97.7%) patients regard methadone as being dangerous. (28.2%). Methadone is most commonly stored in a cupboard (37.9%). All methadone is dispensed in a bottle with a child resistant cap on it. All patients reported they stored their methadone in the original bottle provided by the pharmacist.

**Conclusion:**

Recall of information on safety issues is very poor. Provision of written as well as verbal information is needed. The use of printed safety information cards which patients can take away for future reference may be of use. It is the responsibility of health professionals to ensure they provide information and advice to methadone users on the safe storage of their methadone at home.

## Background

While the safety and efficacy of methadone maintenance treatment has been unequivocally established [1], reports have shown that in the period 1994 to 2004 there were 3298 methadone related deaths in England and Wales [2]. There has however been a significant reduction in these numbers following the introduction of a supervised consumption policy. Methadone has also been described as a 'causal agent in paediatric poisoning over the past decades [3]. It is a sweet green liquid containing the equivalent of 1 mg of morphine per ml. It is therefore very attractive in colour and taste to any child who may be exposed to it in the home. Most patients store methadone at home for at least one day per week which poses serious risks to children who may inadvertently drink the mixture[3,4]

Previous research has shown that only half of patients store methadone in a safe place[5]. It was therefore recommended that all methadone should be prescribed with a measuring device, provided free of charge with each daily dose [6]. At the moment in the UK there is no legal requirement that methadone must be dispensed in child resistant containers. In 2002, an article in the Pharmaceutical Journal recommended that pharmacists should supply methadone in child resistant containers, and always give advice to store it out of the reach of children [7].

Prior to 1999, when the most recent national guidelines in England were published, patients were prescribed take home methadone [8]. Following the publication of these guidelines it is now common- place in the UK to prescribe methadone on a daily basis, with supervised consumption at the pharmacy. Guidelines suggested that daily supervised consumption should be for at least 6 months and often longer [8]. When the patient has demonstrated compliance with treatment, supervised consumption can be gradually discontinued and an increasing number of days supply can be dispensed to take home. Despite the implementation of supervised consumption, most patients must take home methadone on Saturday for unsupervised consumption, as most pharmacies are closed on Sundays.

It is clear that there are a number of safety issues surrounding methadone prescribing. The original audit identified risks to patients and families from the unsafe storage of methadone at home[4]. It was found that recall of provision of information on safety issues was poor. The audit suggested improvements in the information giving process by adopting a standard policy.

The aim of this project was to re-audit the information provided to individuals on a methadone prescription on safe storage of methadone in an outpatient prescribing service. The study audited the extent of information giving and the patients' acceptance of the advice. It also evaluated the impact of the changes suggested by the original audit.

## Methods

### Criteria

The following criteria for the adequacy of information provision were selected after reviewing the criteria in the original audit and after review of the literature.

1. All methadone should be dispensed in a child resistant container when prescribed for home consumption.

2. All patients prescribed methadone should recall being given information on its safe storage.

3. All patients with methadone at home should store it in a safe locked location in a container with child resistant caps.

4. All patients should be aware of the particular risks to children, especially if they have children at home or visiting the home.

5. All patients should be aware of the dangers of methadone use.

### Standards

1. Criteria 1–5 were given a 100% standard.

### Development of survey tools

An audit questionnaire on safe storage of methadone was devised and piloted using a sample of 25 patients, modification to the questionnaire were made based on user feedback and issues identified by the audit team. A copy of the questionnaire administered is available from the corresponding author.

### Setting

The questionnaire was carried out at the Edward Myers Centre, the adult addiction service at the Harplands NHS Hospital in North Staffordshire. The methadone outpatient clinic serves a population of 490,000. At the clinic, individuals on a methadone prescription attend on a weekly basis to provide a urine sample and pick up a prescription for their weekly supply of methadone.

### Administration

An opportunity sample of patients attending the clinic over a period of 7 days was invited to complete the administered questionnaire which contained no patient identifiable information. Each questionnaire took two minutes to complete.

### Subjects

#### Patient survey

In total, one hundred and seventy four patients completed a questionnaire.

#### Documentation survey

Following the publication of the original audit, new documentation was introduced which patients complete when they start the methadone programme. This documentation allows the staff to record that they have given information about how to safely store methadone to each patient and for the patient to sign that they had been given the information. A 10% (40) sample of sets of patients' notes was audited to note whether information had been given.

The documentation was checked to evaluate whether both staff and the patient had signed in the appropriate section of the documentation to say that safe storage information had been provided.

#### Pharmacists' survey

A telephone survey of local pharmacists who dispense methadone in the local area was undertaken. Thirty pharmacists were contacted, of which twenty eight were dispensing to patients during the period of this study. All twenty eight (100%) pharmacists agreed to complete the telephone survey.

### Data analysis

Descriptive statistics were used to analyse the responses to the questionnaire.

## Results

### Response rate

Over a period of one and a half weeks, patients attending a methadone outpatient clinic were invited to complete a short questionnaire. Over 600 patients attend the clinic on a weekly basis. 67 % are males and 33% are females. During the study period, 179 randomly selected patients were approached to complete the questionnaire, only five patients refused, making the response rate 97%. In total, 174 patients completed the questionnaire, 109 (62.6 %) were males, and 65 (37.4 %) were females, 14 (8%) of whom were pregnant.

### Volume of methadone stored at home

In order to ascertain the average volume of methadone that patients may have stored in their homes at any one time. Patients were asked how much methadone they were prescribed each day, as well as how often they picked it up. The volume each user would potentially be storing at home at any one time was calculated.

The mean daily dose of methadone for the 174 patients was 62 mls. (Range 10 mls to 135 mls, SD 22.42). Methadone is prescribed in instalments, 159 (91.4%) patients reported that they pick up their methadone on a daily basis, 145 of these were having supervised consumption. 2 (1.1%) patients reported pick up every two days, 10 (5.7%) twice per week whilst 3 (1.7%) reported 'Other'. Other included one patient who collected their methadone fortnightly, and two patients who collected three times a week.

**Figure 1:**
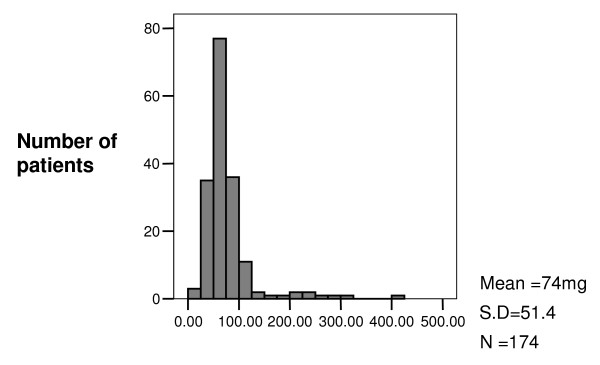
**Volume of methadone stored at home**. Volume of methadone (Img/1 ml) stored (mls).

The volume of methadone stored at home was calculated from the frequency and volume results. As much as 405 mls of methadone is stored at one patients house at one time, however the figure 1 demonstrates that only a small number of patients store large volumes (> 100 mls) at home.

The mean volume stored at home was 74 mls (Range 10 mls to 405 mls, SD 51.38). It must be remembered that as little as 10 mls of methadone has been known to kill children^3^. Therefore a large proportion of the population, are storing potentially fatal doses of methadone at home.

### Location of storage

The most common place of storage was found to be a cupboard. 66 (37.9%) patients reported storage in a cupboard, 50 (28.7%) stored it in the fridge, and 31 (17.8%) reported 'Other'. Table 1 shows the responses reported by the patients who selected their storage location as 'other'.

**Table 1:** Location of storage of methadone in those patients who responded to the option 'other'.

**Location**	**Number of patients**	**Percentage of patients**
On the side	7	22.58
Bedroom drawer	5	16.13
Bedroom	5	16.13
Wardrobe	3	9.68
Table	3	9.68
Mum's House	2	6.45
Bag	2	6.45
Car	1	3.22
Top of stairs	1	3.22
Living Room	1	3.22
In a safe	1	3.22

Total	**31**	**100**

If a patient responded that they stored their methadone in a cupboard or cabinet, they were then asked the further question; is it locked or unlocked? The questionnaire showed that 21 (12.1%) patients kept their methadone in a locked location; the remaining 153 (87.9%) patients stored it in an unlocked location.

### Methadone storage container

All of the 174 patients (100%) who completed the questionnaire reported that they stored their methadone in the original container that the pharmacist provided the methadone in. All 174 (100%) patients also reported that the container which the pharmacist provided had a child resistant cap on it.

### Advice on safe storage of methadone

125 (71.8%) patients who completed the questionnaire had no recall of ever being given information about where they should safely store their methadone. Of the 49 (28.2%) who did recall being given advice, 24 (13.8%) reported it had been given by the clinic, 18 (10.3%) by the pharmacy, 1 (0.6%) by a drug agency and 6 (3.4%) by other sources.

### Advice given by the clinic

40 (10%) sets of patients' notes were randomly selected from the population of the methadone clinic. In each set of notes there should be the signature of the patient and a member of staff to acknowledge that advice had been given about the safe storage of their methadone. The records showed that 17 (42.5%) patients were recorded as having been advised, and the remaining 23 (57.5%) had not been recorded as having been told about safe storage.

### Accessibility to children

83 patients (47.7%) reported that they had children at home, or children who visited their home. Children were classed as those under 16 years of age. 166 (95.4%) reported that children would not be able to get hold of their methadone. Of the 83 patients who did have children at home or visiting the home, 8 patients (9.6%) reported that these children would be able to get hold of their methadone.

### Is methadone dangerous?

When patients were asked the question 'In your opinion, is methadone dangerous to you as a user?', 72 (41.4%) patients replied yes, 99 patients (56.9%) replied no, and 3 patients (1.7%) said they did not know. The same question was asked in regard to dangers of methadone to non users. 170 (97.7%) patients replied yes, 2 (1.1%) replied no, and 2 patients (1.1%) said that they did not know.

### Pharmacist survey

28 pharmacists participated in a telephone survey regarding prescribing methadone. 8 (28.6%) pharmacists reported that advice on safe storage of methadone had been given. The pharmacists dispensed for a mean of 20 patients (Range 1 to 50) 26 (92.9%) confirmed that they would provide a measuring device on request. Only 2 (7.1%) provided a measuring device on each attendance. 3 (10.7%) pharmacists reported that an information leaflet about safe storage of methadone is provided when the patient starts the methadone programme. All 28 (100%) pharmacists dispensed the methadone in a medicine bottle with a child resistant cap on it.

### Audit criteria

The dispensing of methadone in containers with child resistant caps was the only criteria which reached a 100% standard. Nearly 100% of patients were aware of the dangers of methadone use for non-users. Those patients who reported that they stored their methadone in either a medicine cabinet or a cupboard were classed as safe locations and therefore they were said to be aware of the risks of methadone to children. In this case this was 47.7% of the sample population.

The performance on criteria 1 to 5 measured against the defined standards are shown in table 2.

**Table 2:** Performance on criteria 1 to 5 measured against the defined standards.

**Criteria**	**Standards**	**Results**
All methadone should be dispensed in a child resistant container when prescribed for home consumption.	100%	100%
All patients prescribed methadone should recall being given information on its safe storage.	100%	28.2%
All patients with methadone at home should store it in a safe locked location in a container with child resistant caps	100%	12.1%
All patients should be aware of the particular risks to children, especially if they have children at home or visiting the home.	100%	47.7%
All patients should be aware of the dangers of methadone use for non-users.	100%	97.7%

## Discussion

The safety of storage of Methadone can be improved by a number of factors:

### 1. Safe storage containers

Pharmacists have a responsibility not only to ensure that any methadone that is prescribed for home consumption is dispensed in a bottle with child resistant caps on it, but also to give advice to ensure that the methadone is stored in a safe place out of the reach of children [7]. This study has shown that all patients and all pharmacists report that methadone is always dispensed with child resistant caps on. This was the only criterion that reached a 100% standard.

### 2. The provision of measuring devices

When the methadone is stored in an inappropriate device such as a graduated baby's feeding bottle IT can pose a risk to children [9]. The provision of measuring devices is intended to reduce the use of non-standard containers to measure out methadone. When pharmacists were questioned as to whether a measuring device was provided with each prescription of methadone, only 2 (7.1%) provided on every occasion, although 26 (92.9%) said they would provide some form of measuring device on request.

### 3. Provision of information on safety issues

Only 8 (28.6%) pharmacists reported giving information about safe storage. 3 (10.7%) pharmacists reported that the patients sign a contract with them when they begin on the methadone maintenance programme, and this is used as an opportunity to be given verbal advice and also leaflets concerning safe storage. Other pharmacists commented that it is up to the individual pharmacist whether they give advice. Some said they would be more inclined to warn of the dangers to children if the patient came to the pharmacy with a child. One pharmacist commented that all bottles have a label which states it must be kept out of the reach of children so they felt they did not need to re-iterate it.

This study found that of the 18 (10.3%) patients who reported that pharmacists had given them information about where to store methadone, 3 (16.7%) said that they were advised to keep it in the fridge. In total, 50 (28.7%) patients reported that they stored their methadone in the fridge, which is the second most common place of storage after a cupboard. The remaining 47 (94%) patients kept it in the fridge because they preferred to take it when it was cold while others reported that 'you just know it's supposed to go in the fridge'. Patients seem unaware of the fact that keeping methadone in the fridge makes it very accessible to children, increasing the likelihood of a child consuming it.

The best practice guidelines from the Royal Pharmaceutical Society of Great Britain states that 'pharmacists or other appropriately trained pharmacy staff should provide direct input wherever possible to promote harm reduction' [10]. This audit suggests that is not happening in a large number of cases.

### 4. Improving recall of information

Following the original audit which showed that there was poor recall of provision of information about safe storage, it was proposed that new documentation would allow patients to be given verbal advice to store their methadone safely when they commence their treatment. It was then hoped that this would be re-enforced if patients went from supervised consumption at the pharmacy to unsupervised home consumption programmes. Ideally all patients would recall being given information by the clinic staff it and/or the pharmacist.

It was found that despite these new procedures 125 (71.8%) patients did not recall being given any information. Of those who did recall being told to store it safely, 24 (13.8%) reported that the methadone clinic had told them, and 6 (3.4%) answered 'others'. Of the 6 patients who replied "others", 4 stated it was their friends who told them where it should be stored, and the remaining 2 said their partner had told them.

#### Limitations

The patients were sampled randomly, some of these patients may have been on methadone for many years, and some may have only just started. For those who have been on methadone for many years, it is understandable that they may not remember if anybody had ever told them about safe storage.

## Conclusion

This project has demonstrated that there are still a number of serious concerns regarding storage of methadone in the home. It would appear that despite the introduction of a protocol to ensure that staff and patients recorded the fact that safety information had been given, this had only been recorded in 42.5% of notes sampled.

It would be unrealistic and unhelpful for a patient to be educated about storage of their methadone every time they pick it up, however it seems reasonable that at the start of the programme, every few months and if they ever change to being unsupervised they should be reminded. This should be done by all health professionals involved in the care of these patients, including clinic staff, pharmacists, local drug agencies and GPs using verbal and written material or by the use of targeted text messages for those who have mobile phones.

## Competing interests

The authors declare that they have no competing interests.

## Authors' contributions

AM is a 4^th ^Year Medical Student who completed this audit as a special study module option, she devised the questionnaire, collected and analysed the data and wrote the initial draft of the manuscript.

RJM supervised the data collection and facilitated access to the patients

DJW provided clinical supervision to AM and is the responsible clinician for the patient group.

IBC supervised the data analysis, co- supervised AM during the project and during the preparation of the initial draft manuscript.

RNB conceived of the audit, devised the methodology and was the academic supervisor for AM and wrote the final manuscript.

All authors read and approved the manuscript
